# Person Recognition System Based on a Combination of Body Images from Visible Light and Thermal Cameras

**DOI:** 10.3390/s17030605

**Published:** 2017-03-16

**Authors:** Dat Tien Nguyen, Hyung Gil Hong, Ki Wan Kim, Kang Ryoung Park

**Affiliations:** Division of Electronics and Electrical Engineering, Dongguk University, 30 Pildong-ro 1-gil, Jung-gu, Seoul 100-715, Korea; nguyentiendat@dongguk.edu (D.T.N.); hell@dongguk.edu (H.G.H.); yawara18@hotmail.com (K.W.K.)

**Keywords:** person recognition, surveillance systems, visible light and thermal cameras, histogram of oriented gradients, convolutional neural network

## Abstract

The human body contains identity information that can be used for the person recognition (verification/recognition) problem. In this paper, we propose a person recognition method using the information extracted from body images. Our research is novel in the following three ways compared to previous studies. First, we use the images of human body for recognizing individuals. To overcome the limitations of previous studies on body-based person recognition that use only visible light images for recognition, we use human body images captured by two different kinds of camera, including a visible light camera and a thermal camera. The use of two different kinds of body image helps us to reduce the effects of noise, background, and variation in the appearance of a human body. Second, we apply a state-of-the art method, called convolutional neural network (CNN) among various available methods, for image features extraction in order to overcome the limitations of traditional hand-designed image feature extraction methods. Finally, with the extracted image features from body images, the recognition task is performed by measuring the distance between the input and enrolled samples. The experimental results show that the proposed method is efficient for enhancing recognition accuracy compared to systems that use only visible light or thermal images of the human body.

## 1. Introduction

Recently, with the development of digital systems and the high demand for monitoring and security applications, surveillance systems have been rapidly developed. In the conventional setup, a surveillance system uses one or more cameras to collect sequences of images of an observation scene to automatically monitor the people and/or their actions that appear in the scene. Because of this characteristic, the surveillance system has been widely used in security systems to monitor private houses, workplaces, and public areas, or in business to collect customer information [1,2,3,4,5,6]. In a surveillance system, various image processing algorithms can be implemented to extract information from the observation scene such as the detection of incoming persons, and recognition of their age, gender, and actions. With this information, a surveillance system can perform its tasks. For example, the management surveillance system in a shopping mall can count how many people appear in a shop in a period of time, and measure the shopping trend of people according to their age or gender; the surveillance security system can measure types of people’s actions to detect illegal actions if they appear. With more requirements on the surveillance system, the system may be required to recognize an incoming person. During business hours, the shop owner may need to estimate how many times a specific person visits the shop to evaluate the satisfaction of the customers who shop there; the security system in a private house may need to detect strange incoming persons to prevent burglary or other crimes. Therefore, person recognition (verification/identification) capability is required in surveillance systems for advanced tasks.

Recognizing an individual is an important task in many application systems such as the check-in system in a company or government office or the immigration system in an airport. Traditionally, these systems recognize an individual using either a token-based method (such as a key or passwords) or biometric methods (that use the individual’s physical characteristics such as the face [7,8], finger-vein [9], fingerprint [10], or iris patterns [11,12] for recognition). Even though biometric features have proven to be more sufficient in recognizing persons in security systems because of biometric patterns’ advantages of being hard to steal and hard to fake [13], these kinds of biometric features require the cooperation of users and a short capturing distance (z-distance) between camera and user during the image acquisition stage. In addition, these kinds of biometric features are normally poor in quality (small blurred faces or occluded face region) or do not appear (finger-vein, fingerprint, and iris) in captured images in the surveillance system. As a result, these kinds of biometric features are not sufficient to be used in surveillance systems for the person recognition problem. 

Fortunately, the human body contains identity information [14,15,16,17,18,19,20,21,22,23,24,25,26,27,28], and this characteristic can be used for gait-based person recognition in a surveillance system. The clue is that we can roughly recognize a familiar individual by perceiving his/her body shape. Using this characteristic, many previous studies have successfully recognized a person in a surveillance environment using body gait images [21,23,24,27]. In detail, visible light images of the human body are first captured using a visible light camera. With this kind of image, the human body regions are detected and used for identification. For the identification procedure, most of the previous research focused on two main steps for person identification, including optimal image features extraction and distance (similarity) measurement.

Recently, the deep learning framework was introduced as the most suitable method for the image classification and image features extraction problems. Many previous studies have demonstrated that they successfully solved many kinds of problems in image processing systems using the deep learning method. For example, one of the first studies that successfully used deep learning for the recognition problem was the application of a convolutional neural network (CNN) on the handwriting recognition problem [29]. Later, various image processing systems such as face recognition [8], person re-identification [14,15,25], gaze estimation [30], face detection [31], eye tracking [32], and lane detection [33] were solved by using a deep learning framework with high performance. For the body-based person identification problem, the deep learning method was also invoked and produced better identification performance compared to traditional methods. For example, Ahmed et al. designed a CNN network to extract image features using visible light images of the human body [14]. Most recently, Cheng et al. designed a CNN network that can efficiently not only extract the image features, but also learn the distance measurement metrics [15]. However, the use of body images for person identification faces many difficulties that can cause performance degradation in identification systems [15]. There are two main reasons for this problem. First, human body images contain dramatic variations in appearance because of the differences in clothing and accessories, the background, body pose, and camera viewpoint. Second, different persons can share a similar gait and human body appearance (intra-class similarity). In addition, as shown in the above explanation, most previous studies use only visible light images for identification. This approach has a limitation in that the captured images contain both of the above difficulties. In addition, the surveillance system can work only in the daytime because it uses only visible light images. As proved by some previous researches [34,35,36,37], the combination of visible light and thermal images can be used to enhance the performance of several image-based systems such as pedestrian detection, disguise detection and face recognition. As claimed by these researches, the thermal images can be used as an alternative to visible images and offer some advantages such as the robustness to the change of illumination and dark environments. In addition, the detection of humans in surveillance systems was well developed by previous research [38]. This gives us the chance to extract more information using human body images in a surveillance system such as the gender and identity information. 

In order to reduce the limitations of previous studies, in this paper, we propose a person recognition method for surveillance systems using human body images from two different sources: images from a visible light camera that captures the appearance of the human body using visible light, and a thermal camera that captures the appearance of the human body using infrared light that is emitted from the human body by body heat. By using a thermal camera, we can significantly reduce the effects of noise and variation in background, clothing, and accessories on human body images. Moreover, the use of a thermal camera can help enable the surveillance system to work in low-illumination conditions such as at nighttime or in the rain. In addition, we apply a state-of-the art method, called CNN among various available inference techniques, such as dynamic Bayesian networks with ability for adaptation and learning, for image features extraction in order to overcome the limitations of traditional hand-designed image feature extraction methods.

In Table 1, we summarize the previous studies on body-based person recognition (verification/identification) in comparison with our proposed method.

The remainder of this paper is structured as follows: In Section 2, we describe the proposed person recognition method based on the combination of visible light and thermal images of the human body. Using the proposed method, we perform various experiments to evaluate the performance of the proposed method, and the experimental results are discussed in Section 3. Finally, we present the conclusions of our present study in Section 4.

## 2. Proposed Method for Person Recognition Using Visible Light and Thermal Images of the Human Body

### 2.1. Overview of the Proposed Method

**Figure 1:**
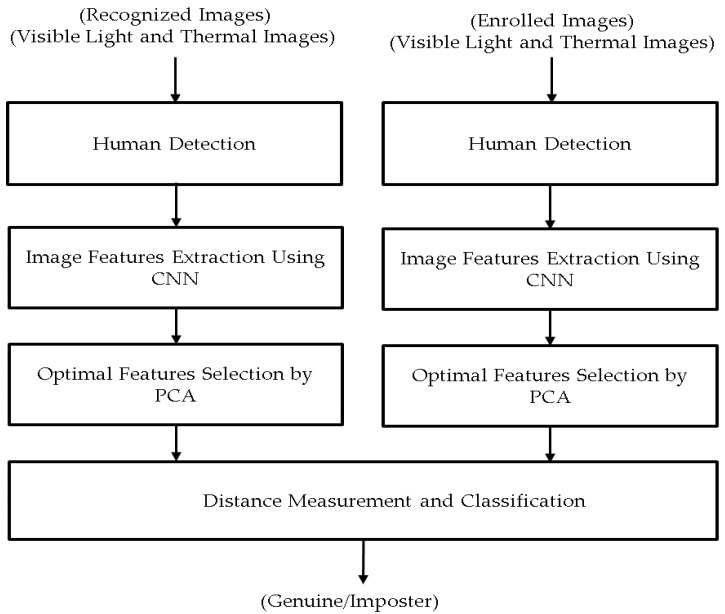
Overall procedure of proposed method for person recognition.

As mentioned in the previous section, our research is intended to recognize a person who appears in the observation scene of a surveillance system. The overall procedure of our proposed method is depicted in Figure 1. As shown in Figure 1, in order to recognize a human in the observation scene of a surveillance system, we first capture the images using two different cameras, including a visible light and a thermal camera. As a result, we capture two images, visible light and thermal images, of the observation scene at the same time. As the processing step of our proposed method, the human detection method is applied to detect and localize the region of a human if it exists in the observation scene. Because of the use of both visible light and thermal images, we enhance the performance of the human detection step by using the detection method proposed by Lee et al. [34]. As proved in this research, the use of both visible light and thermal images can help to enhance the detection performance in various unconstrained capturing conditions, such as different times of day (morning, afternoon, night) or environmental conditions (rainy day) [34].

As the next step in our proposed method, the detection result of the human body (called the human body image) is fed to the feature extraction method to extract sufficient image features for the recognition step. Traditionally, this step plays a highly important role in the recognition performance of the system. Recently, the deep-learning framework has received much attention as a powerful method for image classification and image features extraction. Inspired by this method, we use deep learning for the image feature extraction step in our proposed method. In addition, two traditional image features extraction methods, histogram of oriented gradients (HOG) and multi-level local binary patterns (MLBP), are also used for the image features extraction step along with the deep learning method for comparison purposes. Although we extract the image features using state-of-the-art feature extractors (CNN, HOG and MLBP), the extracted features can contain redundant information because of the effects of background and noise. In order to reduce these kinds of negative effects, we further process the extracted features by applying the principal component analysis (PCA) method to reduce the feature dimensions and redundant information. These image features extraction methods are descripted in detail in Section 2.2. 

Using this procedure, we extract the features from both the visible light and thermal images. These features (visible light features and thermal features) are then concatenated together and used to describe the body of the input human. As shown in Figure 1, our proposed method recognizes the human by measuring the distance from the image features of the enrolled person and those of the input (recognized) person. By this method, the distance between human body images of the same person will be smaller than the distance between the human body images of a different person.

### 2.2. Image Feature Extraction

Image features extraction is an important step that can predict the performance of every recognition/identification system. In our study, we employ two popular traditional hand-designed image features extraction methods, HOG and MLBP, and an up-to-date feature extraction method based on CNN. On the basis of these feature extraction methods, we performed experiments to evaluate the ability of each method to describe the images by measuring the verification/identification accuracy, as described in Section 3.

#### 2.2.1. Histogram of Oriented Gradients

**Figure 2:**
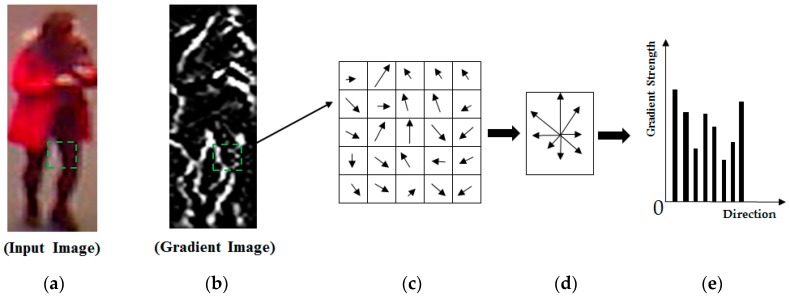
Methodology of image features extraction using the histogram of oriented gradients (HOG) method: (**a**) input image with a given sub-block; (**b**) the gradient image of (**a**); (**c**) the gradient map of the green sub-block in (**a**,**b**); (**d**) the accumulated strength and direction information of the gradient at every pixel in the green sub-block; and (**e**) the final extracted feature for the green sub-block.

The HOG method is one of the most popular methods for describing human body images. This method has been successfully applied to many computer vision problems using human body or face images, such as the pedestrian detection [39], age estimation [40], face recognition [41], gender recognition [42,43]. The principle of the HOG method is that the HOG method constructs histogram features of a sub-block of an image by accumulating the strength and direction of the gradient information at every pixel inside the sub-block. For demonstration purposes, Figure 2 shows the principle of image features formation from a sub-block in an image. As shown in this figure, the gradient information at every pixel inside a sub-block in the horizontal and vertical directions is first calculated. From this information, the strength and direction of the gradient are obtained as shown in Figure 2c. In the final step, this method groups the gradient directions at every pixel into several direction bins and accumulates the gradient strength to form the final histogram feature as shown in Figure 2d–e.

**Figure 3:**
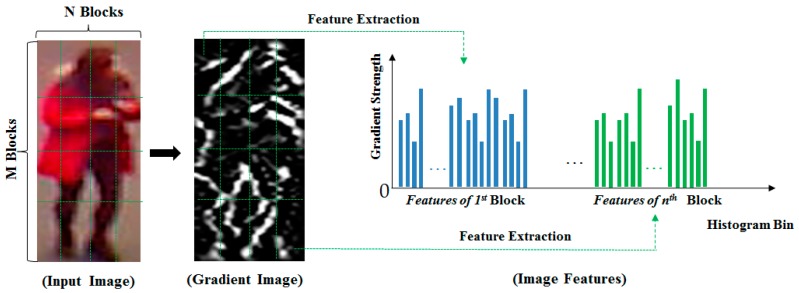
HOG image features formation from a human body image.

In order to extract the image features from an entire image, the image is first divided into *n* (*n* = M × N in Figure 3) overlapping sub-blocks. These sub-blocks are then used to extract the histogram features as shown in Figure 2. As a result, we obtain *n* histogram features corresponding to *n* sub-blocks in the image. The final image features are then formed by concatenating the histogram features of all sub-blocks in the image as shown in Figure 3. In this figure, M and N indicate the number of sub-blocks in the vertical and horizontal directions of the input image.

#### 2.2.2. Multi-Level Local Binary Patterns 

Recently, the local binary pattern (LBP) method has become a powerful image feature extraction method. As proven through various previous studies [7,44,45,46], this method offers the illumination and rotation invariance characteristics for the extracted image features. As a result, this method has been successfully used in many image processing systems such as face recognition [7], gender recognition [44], and age estimation [45,46]. Mathematically, the LBP method extracts a descriptor for each pixel in an image using Equation (1). In this equation, *R* and *P* indicate the radius and the length in bits of the descriptor. The *g_c_* and *g_i_* indicate the gray level of the center pixel and the surrounding pixels that lie on the circle with radius of *R*. As shown in this equation, the descriptor of a pixel is a number that is formed by comparing the surrounding pixels with the center pixel. With this formula, the extracted descriptor of a pixel remains even if the lighting condition is changed (invariant to the illumination characteristic), and the extracted descriptor depends only on the image texture at the small region around the center pixel. In order to extract the image features of an image, the LBP descriptors at all image pixels are first classified into uniform and non-uniform patterns. The uniform patterns are those that contain at most two bit-wise changes from 0 to 1 or 1 to 0; the non-uniform patterns are the remaining ones that contain more than two bit-wise changes from 0 to 1 or 1 to 0. The uniform patterns normally describe good image texture features such as line, corner and spot, whereas the non-uniform patterns are the patterns with associated noise. Therefore, this classification step helps to reduce the effect of noise on the extracted image features. From the classified uniform and non-uniform patterns, the image feature vector is formed by accumulating a histogram of uniform and non-uniform patterns over the entire image. Inspired by the research of Nguyen et al. [46] and Lee et al. [7], we use multi-level local binary pattern (MLBP) to extract the image features of a given image. The difference between LBP and MLBP is that the MLBP features are obtained by dividing the image into sub-blocks with different sub-block sizes and concatenating the LBP features of all sub-blocks together to form the MLBP feature. Consequently, the MLBP features can capture more rich texture information (both the local and global texture features) than the LBP features [7,45,46].

(1) $${\mathrm{LBP}}_{R,P}=\sum_{i=0}^{P-1}s\left(g_{i}-g_{c}\right)\times 2^{i}\text{ where }s\left(x\right)=\{\begin{array}{c} 1,\;if\;x\geq 0\\ 0,\;if\;x<0 \end{array}$$

**Figure 4:**
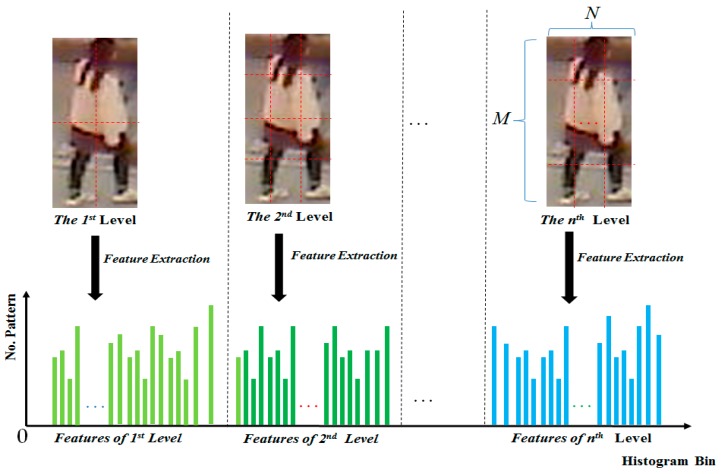
Multi-level local binary pattern (MLBP) image features extraction from a human body image.

For the body-based person verification/identification problem, because the images are captured in the unconstrained environment of a surveillance system, the captured images have problems of large variation of illumination conditions (images can be captured in the morning, afternoon, or night). Therefore, the MLBP method can be used to overcome this problem. In Figure 4, we show a methodology for extracting the MLBP features from input human body images. Using this method, we plan to extract the image texture features that are invariant to changes in illumination conditions.

#### 2.2.3. Convolutional Neural Networks (CNNs)

Recently, deep-learning framework has received much attention in the image understanding and image classification research field. As reported from various previous studies, the deep-learning-based convolutional neural network (CNN) has been successfully applied to various image processing systems such as face recognition [8], handwriting recognition [29], person re-identification [14,15,25], gaze estimation [30], face detection [31], eye tracking [32], and lane detection [33]. This method offers several advantages compared to traditional image recognition methods. First, given that the deep-learning method is constructed by simulating the working of the human brain using convolution operations and neural networks, the deep-learning method can learn and recognize the images in the same manner as a human. Second, unlike the traditional image feature extraction methods such as HOG, MLBP, Gabor filtering, scale-invariant feature transform (SIFT), and speed-up robust feature (SURF), which have a fixed design and parameters for all problems, the deep-learning method has a flexible method for extracting the image features based on a learning method. Using a large amount of training data that demonstrate a specific problem, the deep-learning method performs a learning method to learn the filters that will be used to extract the image features. Because of the learning procedure, the filters that are used for image feature extraction are optimal and suitable for the given problem. In addition, the use of a down-sampling layer makes the deep-learning slightly invariant to the misalignment of images, and image normalization makes the deep-learning invariant to changes in illumination conditions.

**Figure 5:**
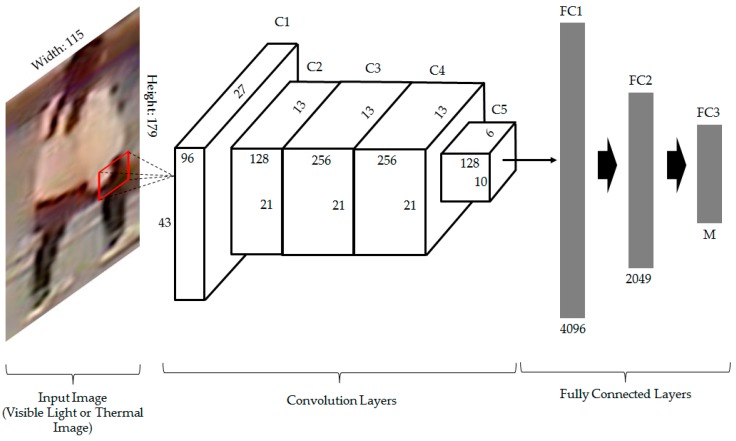
The designed convolutional neural network (CNN) structure for person recognition in our proposed method.

Essentially, the CNN consists of two main components: convolution layers and fully connected layers [8,29]. Of the two components, the convolution layers undertake the image feature extraction, and the fully connected layers classify the images on the basis of the extracted image features. To extract the image features, the CNN method uses a large number of filters with different sizes at several convolution layers followed by the pooling layers. The main advantage of CNN is offered at this stage by which all the filters (filter coefficients) are learned using training data. The efficiency of the CNN network depends on the depth of the network (the number of convolution and fully connected layers) [47]. Inspired by this method, we designed and trained our CNN network for the person recognition problem as shown in Figure 5. In addition, the detail description of the CNN network in Figure 5 is given in Table 2. In Table 2, M indicates the number of individuals in the training database, and ReLU indicates the rectified linear unit.

As shown in Figure 5, our CNN structure contains five convolution layers and three fully connected layers. In this figure, C1~C5 indicate convolution layer 1 to convolution layer 5, and FC1~FC3 indicate fully connected layer 1 to fully connected layer 3. As a preprocessing step, the human body images are scaled to 115 × 179 pixels in the horizontal and vertical directions before being fed to our CNN structure. In the training stage, the training images are fed to the network to learn the filter coefficients and the weights of the fully connected layers. As a result, the trained CNN model that contains all the filter coefficients and weights of the fully connected layers are stored in memory to use in the testing stage. Because we use two different kinds of input images (visible light and thermal images), the training is performed two times, once with only visible light images and once with only thermal images. As shown in Figure 1, the CNN models are used to extract the image features that is then used to measure the distance between images. Therefore, in the testing stage, we use the trained CNN model to extract the image features of testing images. For this purpose, we use the output features at the second fully connected layer. As a result, we obtain a feature vector of 2048 components (a vector in 2048-dimensional space) for each input visible light or thermal image.

In our study, we focus on the body-based person recognition/identification problem. As our experiments, the height and width of human body images are not quite similar. Normally, the height is about from 1.5 to 2.0 times larger than the width because of the natural shape of human body. If we try to represent the human body image as a square shape, it is so stretched in the horizontal direction compared to vertical one that the important information about the body shape can disappear due to image distortion. As an alternative, we can use the square shape without image stretching, but additional information about the background at the left and right area of the human body can be included in the image of the square shape, which can cause the degradation of person recognition by CNN. 

In addition, the size of the human body images is also smaller than 224 or 227 pixels because of the far distance of our image capturing system considering the conventional surveillance environment. Although we can design our CNN architecture to use the input images in size of 224-by-224 or 227-by-227 pixels that are similar to previous researches in [47,48], it can increase the processing time and memory usage of a recognition system by CNN. Therefore, we design the input images as 115 pixels in width and 179 pixels in height that are similar to the original size of the human body images in our experiments.

#### 2.2.4. Optimal Feature Extraction by Principal Component Analysis and Distance Measurement

The human body images contain large variation because of the capturing conditions, the random appearance of clothes and accessories, and the negative effects of the background. As a result, the extracted image features can contain redundant information. To reduce the effects of redundant information, we apply the principal component analysis (PCA) method on the extracted features [43,45].

In the final step of our proposed method, the similarity between images is measured to recognize the human by calculating the distance between image feature vectors as depicted in Figure 1. As mentioned in the previous section, the output of the feature extraction step is an image feature vector in the form of a histogram feature. As a result, we will use the two different histogram distance measurements to measure the similarity between two image feature vectors, including the Euclidean distance (as shown in Equation (2)) and cosine distance (correlation distance, as shown in Equation (3)):

(2) $$d\left(H_{1},H_{2}\right)=\sqrt{\underset{i}{\sum }{\left(H_{1}\left(i\right)-H_{2}\left(i\right)\right)}^{2}}$$

(3) $$d\left(H_{1},H_{2}\right)=\frac{\sum_{i}\left(H_{1}\left(i\right)-\bar{H_{1}}\right)\left(H_{2}\left(i\right)-\bar{H_{2}}\right)}{\sqrt{\sum_{i}{\left(H_{1}\left(i\right)-\bar{H_{1}}\right)}^{2}\sum_{i}{\left(H_{2}\left(i\right)-\bar{H_{2}}\right)}^{2}}}$$

In Equation (3), the average histogram $\bar{H_{k}}$ is defined as $\bar{H_{k}}=\frac{1}{N}\underset{i}{\sum }H_{k}\left(i\right)$ and N is the number of histogram bins of image features. Using the distance measurement method in Equation (2) or (3), we can measure the similarity between the two image features. 

## 3. Experimental Results

### 3.1. Description of Database and Performance Measurement

**Figure 6:**
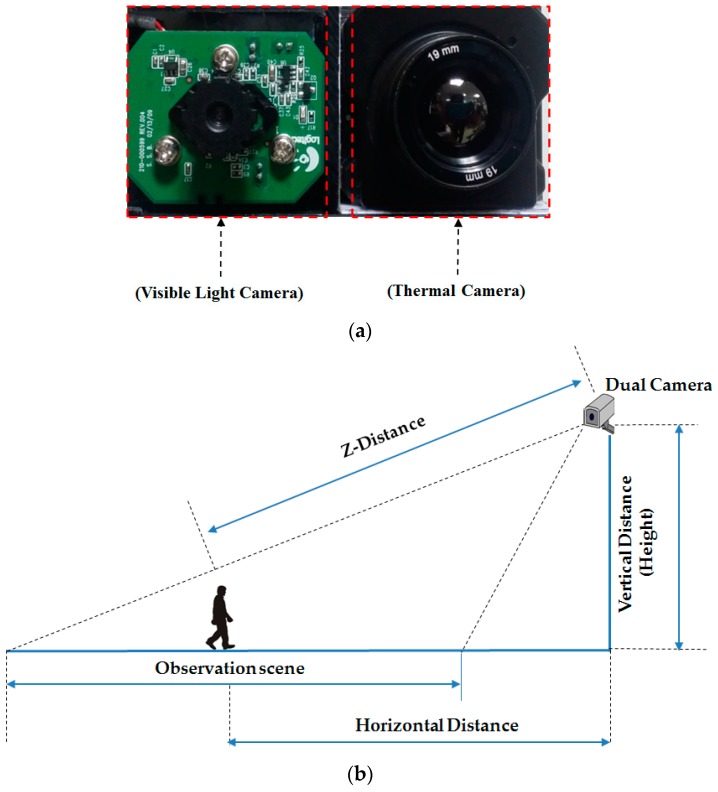
Dual visible light and thermal camera and experimental setup for data acquisition in our study: (**a**) the dual visible light and thermal camera; and (**b**) the experimental setup.

Although there are several public databases for person identification research such as the CUHK01 [49] and CUHK03 databases [50], the VIPeR database [51], the *i*LIDS-VID database [52], and the PRID2011 database [53], these databases cannot be used in our research because they contain only visible light images. Therefore, to evaluate the performance of our proposed method for person identification, we established a new database by capturing the visible light and thermal images of human body at the same time using a dual visible light and thermal camera as shown in Figure 6a. In Figure 6b we show the experimental setup for data acquisition. In our dual camera installation, the visible light images are captured using a visible light webcam camera (C600, Logitech, Lausanne, Switzerland) [54]; and the thermal images are captured using the Tau2 camera (FLIR systems, Wilsonville, OR, USA) [55]. These two kinds of camera are rigidly attached closely together on a panel as shown in Figure 6a in order to capture the visible light and thermal images without any differences between capturing times. Then, the dual camera was placed on the top of a building approximately 6 m (“Vertical Distance” value in Figure 6b) in height (from the ground) in order to simulate the normal working condition of a surveillance system. 

**Figure 7:**
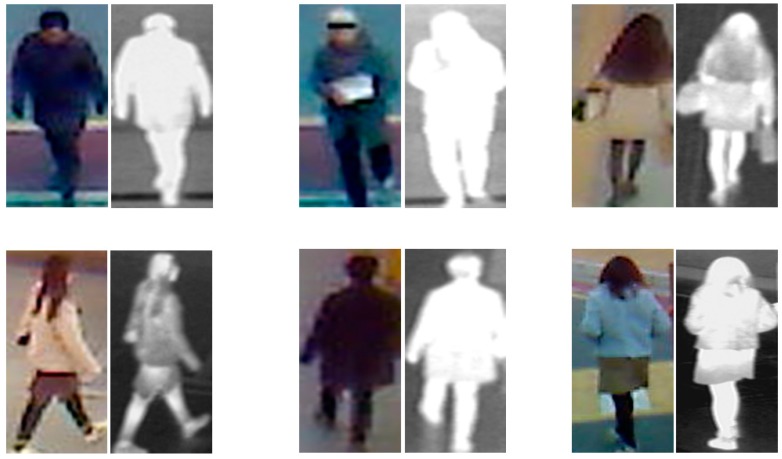
Examples of visible light and thermal image pairs of people in our collected database.

Using the dual camera and experimental setup in Figure 6, we captured an image database of 412 persons while people are moving naturally without any instruction. For each person, we captured 10 visible light images and the corresponding 10 thermal images. Among the 412 persons, there are 254 females and 158 males. In addition, 156 people were captured from the front view and the other 256 people were captured from the back view. Because the images were captured when the people are moving, there exist differences on body-pose, capturing distance, and illumination condition among the 10 images of each person. However, the weather condition, viewing angle of camera, and captured view (front/back view) are same among 10 images of the same person. Consequently, our database contains 4120 visible light images and 4120 corresponding thermal images of 412 different classes. We made our database available for researchers through [56], from which comparisons can be done. Figure 7 shows some example image pairs in our collected database. As shown in this figure, even though the visible light images contain large variation of clothes or background, the thermal images mainly capture the body-pose. This offers the ability for human detection and recognition using thermal images. In detail, as shown in Figure 7, the distinctiveness of body area from background in thermal image is larger than that in visible light image, which can make it easier to detect human region. In addition, the thermal image shows the information of body shape, which enables the rough identity of people based on body shape to be perceived. And, detail texture, color and gray information of clothes disappear in the thermal image, which can make the recognition performance robust to the change of clothes and variation of environmental illumination. Therefore, the thermal image can be used as a complement for visible light images for the person recognition problem. 

Because the deep-learning method requires a training procedure for learning the optimal network’s parameters, we divided the collected database into training and testing databases. In our research, we divided the working database into training and testing sub-databases five times to perform a five-fold cross-validation procedure. For this purpose, we assigned the images of approximately 80% of individuals in our collected database as the training database, and the other images (approximately 20% of the individuals) in our collected database were assigned as the testing database. As a result, the recognition accuracy of the system was evaluated by the average accuracy of five training and testing trials. With the training database, we performed the training procedure by classifying the input images into classes of individuals to learn the CNN network’s parameters. As a result, we obtained a CNN model that well describes the characteristics of each individual in the training database. Because the training database contains a large number of individuals, the trained CNN model can be seen as the optimal model to describe the characteristics of a new input human body image. As mentioned in Section 2.1, the trained model is saved and used to extract the image features for person recognition purpose in our proposed method. 

As proved in previous research [48], the training of a CNN model usually faces the problem of over-fitting due to the large amount of learning parameters and the limitation of training data. Basically, the CNN method requires users to train the network using a huge number of training images, from which the trained CNN model can reflect the characteristics of all training images in various conditions. However, collecting a huge database for training is normally a very hard task. As indicated in this research, the data augmentation and dropout methods are normally used to solve this problem. From the results of this research, we applied these two methods in the CNN training procedure to reduce the over-fitting problem. In detail, the data augmentation is first applied to enlarge the database. Augmented images are artificially generated by image translation and cropping in the left and right horizontal directions, respectively, based on the center position of original image of human body in our database. Additional augmented images are also generated by image translation and cropping in the upper and lower vertical directions, respectively. This scheme of data augmentation has been already used in previous research [48]. Consequently, we can enlarge our database to make it five times larger than the original database with images that contain misalignment due to the boundary pixel removal. The detailed description of the training and testing database (original database and augmented database) are given in Table 3. In addition, the dropout method is also applied in the CNN structure in Figure 5 with the random probability of neuron disconnection (dropout value) of 0.5. 

Normally, the data augmentation procedure is applied on the training database to generalize the training database, and thus, reduce the over-fitting problem. However, we also performed the data augmentation on the test dataset in our experiments to generalize the images in the test dataset. In our system, the input images to CNN for extracting image features are strongly affected by the performance of human region detection method. As a result, incorrect images could be entered to our system. These incorrect images are normally the shifted versions of correct images because of imperfect of human detection algorithm. Therefore, we can make various possible input images through data augmentation method to simulate the operation of real surveillance systems in our experiments. This kind of scheme was already used in previous research based on CNN [32].

As mentioned in Section 2.2.3 and Section 2.2.4, the trained CNN model that resulted from the training procedure will be used to extract the image features of the training and testing databases. These features are further processed by PCA to reduce the effects of noise and the problem of high-dimension image features. The recognition system can operate in two modes: verification mode and identification mode. The difference between the two modes is that the verification mode performs the one-by-one matching, whereas the identification mode performs the one-by-n matching. As a result, the switching between verification mode and identification mode can be done according to purpose of a specific application. In our experiments, we measured the performance of both verification and identification modes to evaluate the efficiency of our proposed method for gender recognition problem. With the extracted image features, the distances between images were measured using Equation (2) (for Euclidean distance) and Equation (3) (for correlation distance), by which the distance between images of the same individual (genuine distances) should be smaller than those of images between different individuals (imposter distance). In order to measure the verification performance of our proposed system, we used the equal error rate (EER) criteria. The EER indicates the case when the false acceptance rate (FAR) is equal to the false rejection rate (FRR). In our case of person verification, the FAR is the error rate when we recognize two images of two different persons as the images of the same person. In contrast, the FRR is the error when we falsely recognize two images of the same person as the images of the two different persons. Normally, the FRR value is re-represented by the genuine acceptance rate (GAR) in verification systems, by which the GAR is calculated by (100-FRR) (%). Therefore, a system with a small value of EER indicates high verification performance (low error). In our experiments, because of the five-fold cross validation procedure, the EER of the system was measured by taking the average EER of five testing databases. For the identification mode, we used the cumulative matching characteristic curve (CMC) for performance measurement. Generally, the CMC curve is a rank-based metric that describes the correct recognition according to the number of acceptable images [14]. Therefore, it is typically used to measure the accuracy of 1-by-n identification systems.

### 3.2. Experimental Results

#### 3.2.1. Optimal Feature Extraction Based on CNN

In our first experiment, we trained the CNN model in Figure 5 using the human-body database described in Table 3. Because the visible light images and thermal images have different characteristics as explained in Section 2.1, we performed the training procedure twice, once using only visible light images and once using only thermal images. For the model initialization, we set the dropout value to 0.5 as suggested in the work by Krizhevsky et al. [48]. The initial values of the filters and weights in the network of Figure 5 were randomly initialized using the Gaussian distribution with zero mean and 0.01 standard deviation, and the number of epochs was 60. As a result, we obtained two CNN models for image features extraction, one for visible light images and one for thermal images. 

**Figure 8:**
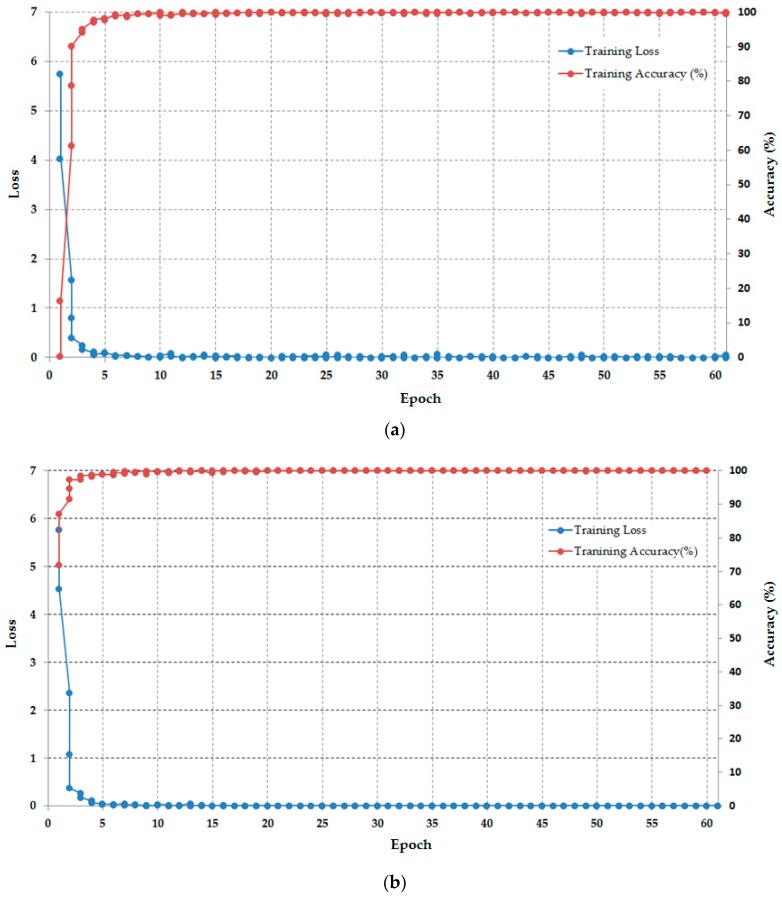
The average convergence graphs of CNN training from five-fold cross-validation across training epochs: (**a**) Using visible light images, and (**b**) Using thermal images.

In order to show the convergence of the CNN training process, we measure the classification accuracies and the loss curves during training across number of epoch using visible and thermal images. Figure 8a shows the average convergence graphs of CNN training from five-fold cross-validation across training epochs using visible light images, and Figure 8b represents those using thermal images. As shown in this figure, at the initial step (epoch is 1), the classification accuracies (of visible and thermal images) are poor because the networks used the initial non-optimal parameters (Gaussian distribution with zero mean and 0.01 of standard deviation). As a result, the losses (of visible and thermal images) are very high. However, after several epochs, the classification accuracies are increased up to 100% and the losses are much reduced (closed to zero). These results means that the network’s parameters are estimated toward optimal ones. From this figure, we can conclude that the training process was performed correctly in learning the network’s parameters to extract the image features and classify the images into group of individuals.

**Figure 9:**
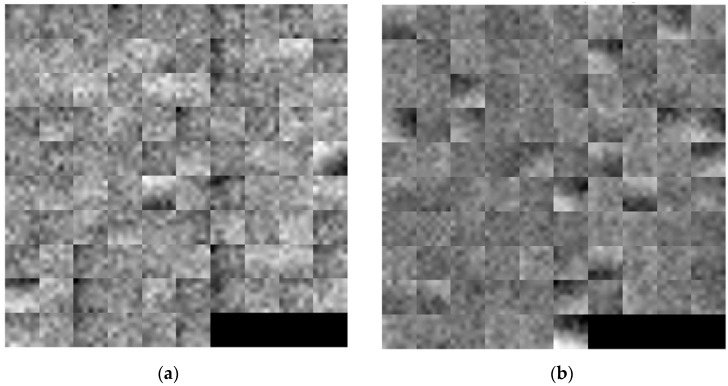
The 96 trained convolution filters in the size of 7 × 7 × 1 obtained in the first convolution layer using our CNN configuration in Figure 5 and our training database: (**a**) the filters obtained using visible light images, and (**b**) the filters obtained using thermal images.

In Figure 9, we show the 96 trained convolution filters obtained at the first convolution layers of the CNN model for visible light images (Figure 9a), and the CNN model for the thermal images (Figure 9b). As shown in this figure, the trained convolution filters are mainly for edge and blob detection. Compared to the results in previous research by Krizhevsky et al. [48] that the trained filters are mainly in the Gabor-like shape, the shapes of the filters in our study are different. The reason is that the working databases used in the two studies are different, and the human-body images obtained in a surveillance environment are normally in poor quality (small and blurred images) and contain simple textures such as lines and blobs.

#### 3.2.2. Experiments Using Euclidean Distance

As mentioned in previous sections, our study exploits the person verification ability of various system configurations using image feature extraction methods (systems using HOG, MLBP, and CNN for feature extraction), distance measurement methods (Euclidean versus correlation distance), and with/without applying PCA for noise and feature dimension reduction. In our next experiments, we first used the Euclidean distance as the distance measurement to measure the similarity between images using image features extracted by the HOG, MLBP, and CNN methods. The other distance measurement based on correlation will be used for the next experiments, described in Section 3.2.3. The Euclidean distance is a highly popular distance measurement method that measures the physical distance between two points in n-dimensional space using Equation (2). In biometrics, the Euclidean distance has also been successfully used in applications such as person recognition [25], finger-vein recognition [57], and face recognition [58]. To measure the verification performance, we first measured the distance between image pairs. Because there exist similarities between images of the same person, the measured distance between two images of the same person tends to be smaller than the measured distance between two images of different persons. From this characteristic, the distance that is smaller than a threshold indicates that the two images are captured from the same person. Otherwise, they are treated as images of different persons. The threshold value is experimentally determined using the training database.

For the first experiment in this section, we measured the verification performance using the raw extracted image features. For this purpose, the image features are extracted by one of three methods (HOG, MLBP, or CNN) and used directly to measure the distance using the Euclidean distance measurement method. For comparison purposes, the verification performance of the three feature extraction methods (HOG, MLBP, and CNN) were measured and compared as shown in Table 4. In Table 4, we show the comparative verification performances of the recognition systems that use Euclidean distance without applying PCA for noise and feature dimension reduction on the three feature extraction methods (HOG, MLBP, and CNN), and three different kinds of images (visible light images, thermal images, and the combination of visible light and thermal images). As shown in this table, using only the visible light images, we obtained an EER of 12.085% using the HOG method, 13.735% using the MLBP method, and 7.315% using the CNN method. These results proved that the CNN method can extract the image features more efficiently than the other two feature extraction methods (HOG and MLBP). Using only the thermal images for verification, we obtained an EER of 13.905% using the HOG method, 16.695% using the MLBP method, and 6.815% using the CNN method. Although the verification errors were still high (about 13.9% and 16.7% using the HOG and MLBP, and 6.8% using CNN), these results demonstrate that the thermal images can be used for the human recognition problem. As explained in Section 1, the previous studies on body-based person recognition have a limitation in that they used only visible light images for recognition. Consequently, their systems have strong effects of noise and the random image textures such as backgrounds or clothes. In order to overcome this limitation, our study combines the visible light images and thermal images to reduce the noise and background effects. The last column in Table 4 indicates the performances of the combinations of visible light and thermal images for verification purposes using the HOG, MLBP, and CNN feature extraction methods. Using the HOG method, we obtained an EER of 11.055%, smaller than that of the system using only visible light images (12.085%) and thermal images (13.905%). Using the MLBP feature extraction method, the EER was reduced from 13.735% using visible light images and 16.695% using thermal images to 12.775% using the combination of the two kinds of images. Finally, the combination of visible light and thermal images of the human body helped to reduce the EER from 7.315% using visible light images and 6.815 % using thermal images to 4.285%. As we can observe from these results, the combination of visible light and thermal images is better than the use of a single kind of image (only visible light or only thermal images) for the verification problem. Through these results, we can see that the combination of visible light images and thermal images is sufficient for the body-based person recognition problem. 

**Figure 10:**
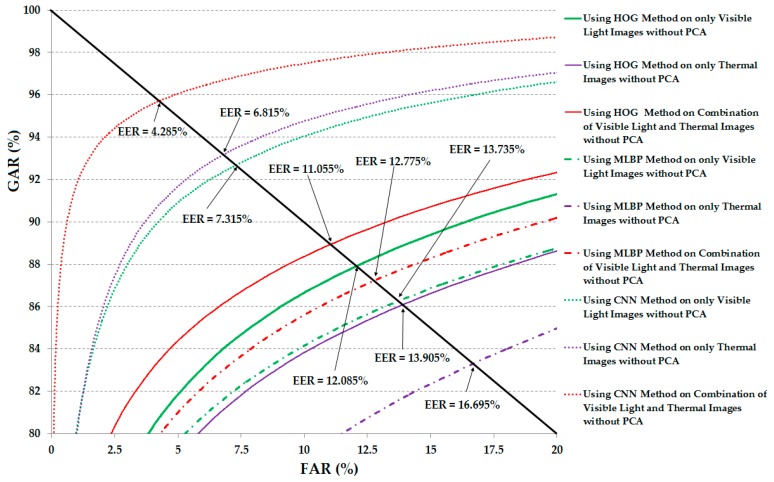
Receiver operating curves (ROC) of the verification systems (system that uses only visible light, only thermal, and a combination of visible light and thermal images for verification) using Euclidean distance without applying principal component analysis (PCA).

For demonstration purposes, Figure 10 shows the receiver operating curves (ROCs) of the verification system using the various system configurations (from Table 4). As shown in this figure, the combination of visible light and thermal images offered better verification results than those of the use of a single kind of human body image in all the cases of feature extraction methods.

**Figure 11:**
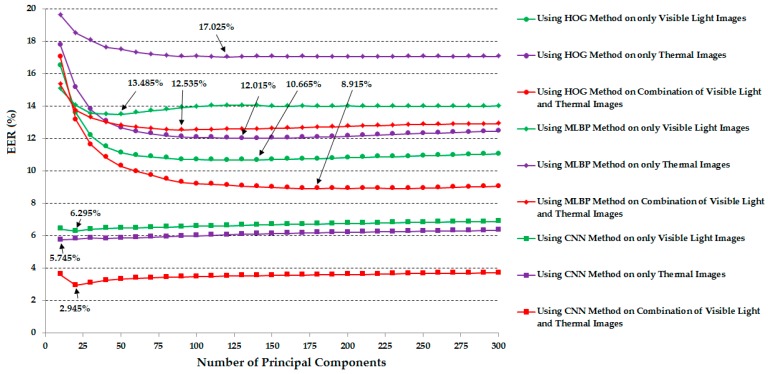
Verification accuracy (EER) of the recognition systems using Euclidean distance according to the number of principal components in PCA.

In these experiments, the extracted image features were used directly for verification purposes. As explained in the previous sections, the use of raw image features has a limitation in that the recognition system can suffer the effects of noise and high-dimension feature vectors. Therefore, we further performed experiments using PCA for noise and feature dimension reduction purposes. The detailed experimental results are shown in Figure 11. In these experiments, we performed the verification using various numbers of principal components in the PCA method (from 10 to 300 in increments of 10). The optimal number of principal components with each system configuration was chosen by which the best verification performance was obtained. The summary verification performance data are given in Table 5. Compared to the verification performance in Table 4, the application of the PCA method on the extracted image features is much better than the case of using the extracted image features directly for the verification problem, especially for the case of the combination of visible light and thermal images using the CNN feature extraction method. In detail, the best verification performance was 2.945% using the combination of visible light and thermal images and the CNN feature. This performance is much smaller than 4.285% in the case without using PCA. In addition, the experimental results in this table again confirm that the combination of visible light and thermal images can produce higher verification accuracy than the use of a single kind of human body image based on visible light images or thermal images. 

**Figure 12:**
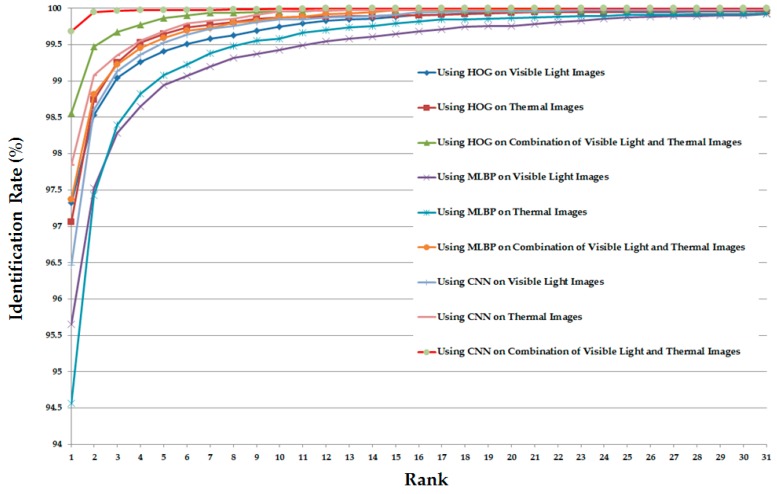
Cumulative matching characteristic curve (CMC) curves using Euclidean distance with various system configurations for the identification problem.

As the final experiment in this section, we measured the CMC of various system configurations (in Table 5) for the identification purpose. The detailed experimental results are shown in Figure 12. As shown in this figure, the CMC curves of the systems that use the combination of visible light and thermal images always offer the better identification results compared to the systems that use only visible light or only thermal images for identification. In addition, the CMC curve of the system that uses the CNN feature extraction method on the combined images (visible light and thermal images) offers the best identification accuracy compared to the others.

#### 3.2.3. Experiments Using Correlation Distance

In Section 3.2.2, we showed experiments that use Euclidean distance to measure the similarity between images. The reason for the use of Euclidean distance is that this similarity measurement method has been used in previous studies [25,57,58]. In this section, we will exploit a new kind of distance measurement method for similarity evaluation based on correlation measurement. As shown in Equation (3) in Section 2.2.4, the correlation method is used to measure the cosine distance between two feature vectors in n-dimensional space. As a result, if the two feature vectors are in the same or a similar direction, the correlation distance is close to one. Otherwise, the correlation measurement varies from zero to one. For human-body image-based recognition, if the two images are captured from the same person, there exist several similarities between them. Consequently, the extracted image features of the two images are two feature vectors that have similar directions. As a result, the measured correlation will be close to one. In the case of two images from two different persons, the extracted image feature vectors can have different directions. Consequently, the measured correlation distance will be close to zero. In order to have consistent meaning with the Euclidean distance that the two similar images should have a small measured distance, we use the inverted score measurement of correlation measurement using Equation (4). In this formula, the *d*(*H*_1_,*H*_2_) indicates the correlation distance measurement between two feature vectors (*H*_1_ and *H*_2_) using Equation (3). Using this formula, the measured correlation distance between two similar images will be small (close to zero), whereas the measured correlation distance between two different images will be much larger than zero.

(4) $$\mathrm{COR}=\left(1-d\left(H_{1},H_{2}\right)\right)$$

**Figure 13:**
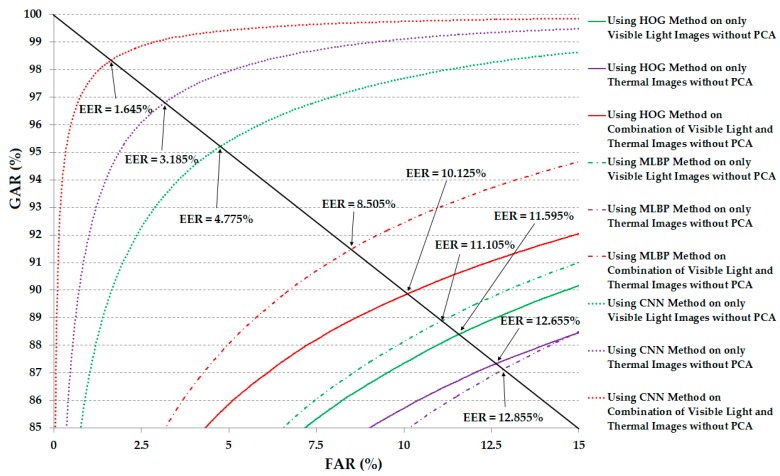
ROC curves of the verification systems (system that uses only visible light, only thermal, and a combination of visible light and thermal images for verification) using correlation distance without applying PCA.

Similar to the experiments in Section 3.2.2, we measured the verification performance of various system configurations using various feature extraction methods (HOG, MLBP, and CNN), three kinds of human body images (visible light, thermal and a combination of visible light and thermal images), and with and without applying the PCA method for noise and image feature dimension reduction using correlation distance in Equation (4). In the first experiment of this section, the systems that do not use the PCA were evaluated. The detailed experimental results are shown in Table 6 for various system configurations. These experimental results are similar to the results given in Table 4 except that the Euclidean distance in Table 4 was replaced by correlation distance in this experiment. As shown in Table 6, the use of the HOG method produced an EER of 11.595% using only visible light images, 12.655% using only thermal images, and 10.125% using the combination of the two. Using the MBLBP method, the verification accuracy (EER) was 11.105%, 12.855% and 9.885% using only visible light images, only thermal images, and the combination of visible light and thermal images, respectively. Compared to the corresponding results in Table 4, we can see that we obtained better verification performance in this experiment using correlation measurement instead of Euclidean distance in Table 4. In particular, by using the CNN method, we obtained EERs of 4.774%, 3.185%, and 1.645% using only visible light images, only thermal images, and a combination of visible light and thermal images, respectively. The best verification accuracy in this experiment was 1.645%, much smaller than 4.285% in case of the system using the CNN feature without PCA in Table 4, and even smaller than 2.945% in the case of the system using the CNN feature with PCA in Table 5. This result indicates that the correlation distance is superior to the Euclidean distance for the verification problem. These results again prove that the combination of visible light and thermal images helps to enhance the verification performance. In Figure 13, we show the ROC curves of the various system configurations. As shown in this figure, the ROC curves of the system that use the combination of visible light and thermal images always show better performance than the use of only visible light images and only thermal images.

**Figure 14:**
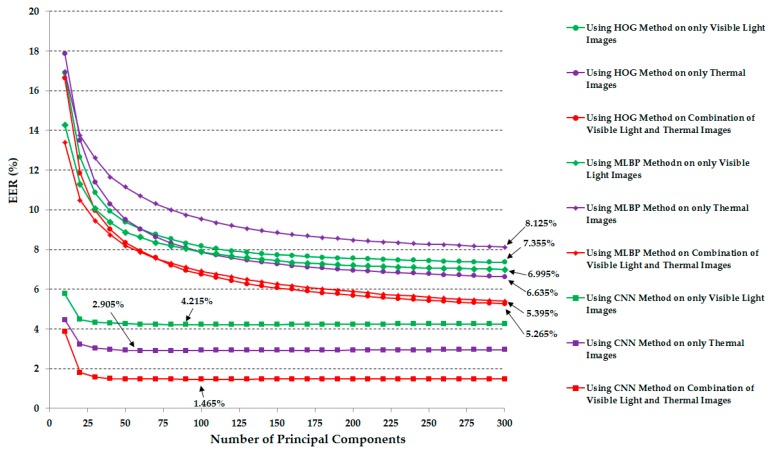
Verification accuracy (EER) of the recognition systems using correlation distance according to the number of principal components in PCA.

As shown in Table 5 and Figure 11, the PCA method helped to enhance the verification performance using Euclidean distance. From these experimental results, in our next experiments, we measured the verification performance of the recognition systems that use the correlation distance and PCA for noise and feature dimension reduction. Similar to our previous experiment in Section 3.2.2, we performed various experiments using all feature extraction methods (HOG, MLBP, and CNN) and various numbers of principal components (from 10 to 300 in increments of 10). In Table 7 we summarize the best verification accuracies (EERs) according to the feature extraction methods and the type of image. In addition, Figure 14 shows the verification accuracies of various system configurations according to the number of principal components. Compared to the results in Table 6 that did not use the PCA method, the use of PCA in this experiment produced better verification accuracies. In detail, using the HOG feature extraction method, we reduced the error from 11.595%, 12.655%, and 10.125% to 7.355%, 6.635% and 5.265% using only visible light images, only thermal images, and a combination of visible light and thermal images, respectively. Using the MLBP feature extraction method, the errors are also reduced to 6.995%, 8.125%, and 5.395%, much smaller than the EERs of 11.105%, 12.855%, and 9.885% in Table 6. However, the verification accuracy using the CNN feature extraction method in this experiment is only slightly enhanced compared to those of Table 6. The best verification accuracy that we obtained in this experiment was 1.465%. This error is also the smallest error in our experiments. Through these experimental results and the previous experimental results given in Table 4 and Table 5, we can conclude that the combination of visible light and thermal images of the human body is efficient for enhancing the verification performance regarding the feature extraction methods and the kind of distance measurement metric. In addition, the CNN feature extraction method outperforms the other methods of HOG and MBLP. These experiments also confirm the advantage of our proposed method compared to previous studies on the human body image-based person recognition problem applied in surveillance systems.

**Figure 15:**
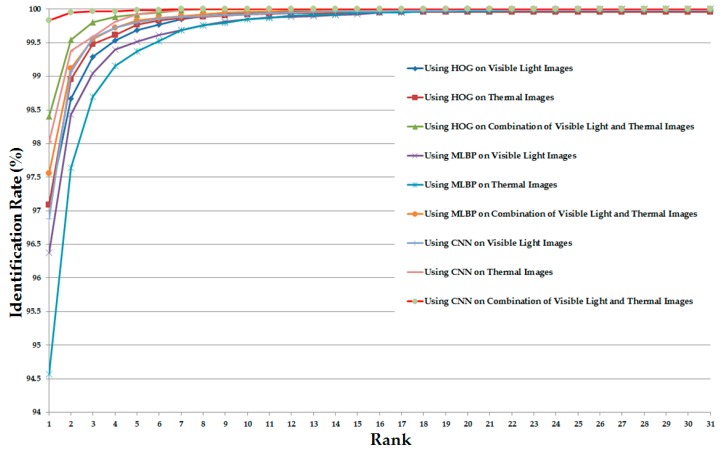
CMC curves using correlation with various system configurations for the identification problem.

Similar to Figure 12 but for the case of the correlation distance measurement instead of Euclidean distance, Figure 15 shows the CMC curves of the various identification system configurations in this experiment. Again, the CMC curves show that the combination of visible light and thermal images can help to enhance the identification accuracy compared to the use of a single kind of human body images (only visible light images or only thermal images).

As explained in Section 3.1, we used our collected database for our experiments because of there are no public databases that contain both visible light and thermal images of human bodies. Unlike previous studies that captured images using two different cameras with non-overlapping observation scenes [49,50,51,52,53], we captured a sequence of human body images in a single view. As a result, the difference between images of the same person in our database is smaller than that of previous databases [49,50,51,52,53]. Therefore, the verification accuracy seems to be higher than those in previous studies (EER of 1.465% in Table 7). However, the contribution of our research is that we focus on the combination of two kinds of images (visible light and thermal images of human body) instead of using only visible light images. Through our various experiments presented in Section 3.2.2 and Section 3.2.3, we conclude that the combination of visible light and thermal images is efficient for enhancing the recognition performance of body-based person recognition systems. Among various system configurations, the system that uses the combination of visible light and thermal images with PCA and correlation distance measurement outperforms the other configurations and produced the best verification accuracy of 1.465% with our collected database.

#### 3.2.4. Part-Based Person Recognition 

**Figure 16:**
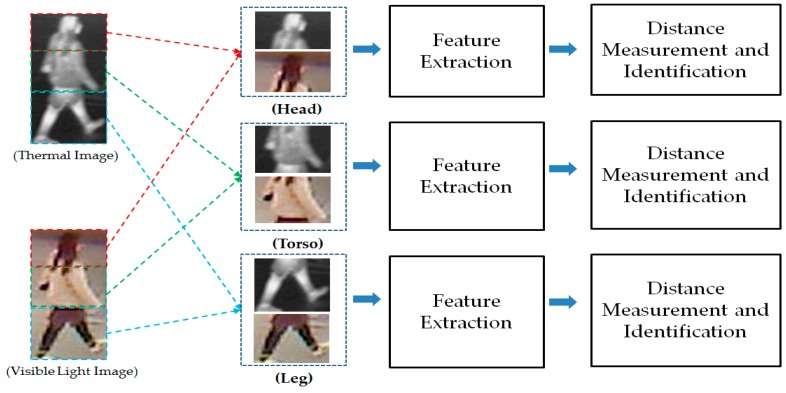
The separated body parts used in our experiments in this section.

**Figure 17:**
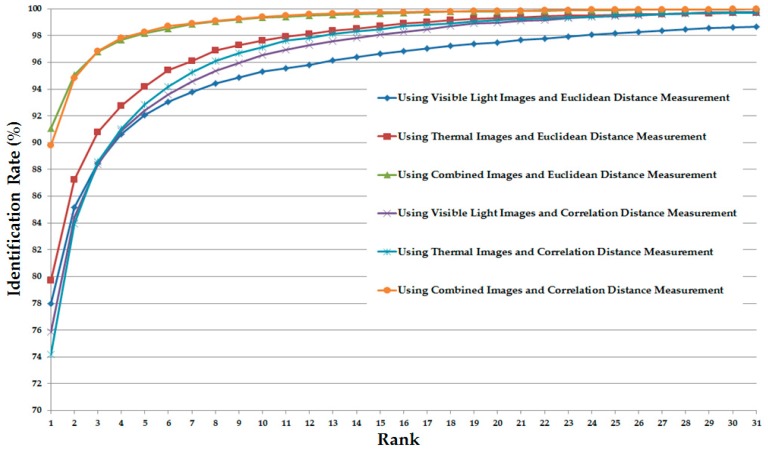
CMC curves of the identification systems that use the head part for the identification problem.

**Figure 18:**
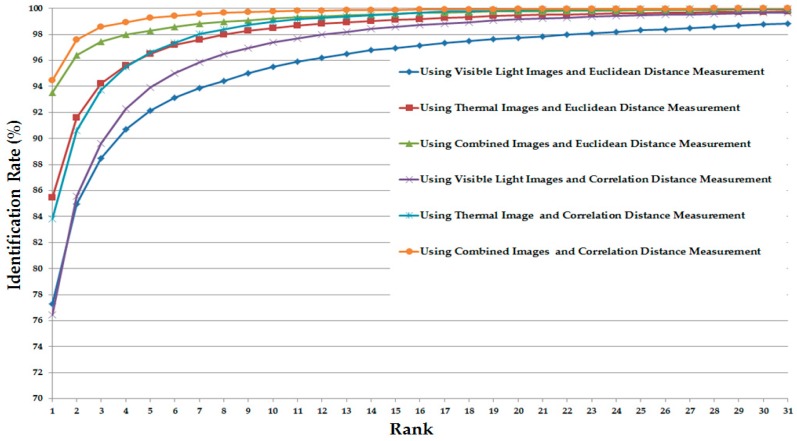
CMC curves of the identification systems that use the torso part for the identification problem.

**Figure 19:**
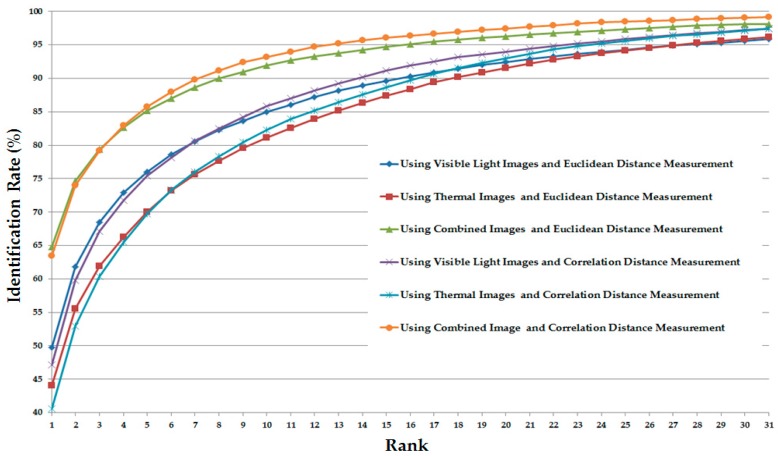
CMC curves of the identification systems that use the leg part for the identification problem.

As our next experiments, we attempted to exploit the recognition ability of different parts of human body images. For this purpose, we divided the human body images into three parts (head, torso, and leg parts) as shown in Figure 16. As a result, we obtained three visible light and thermal pairs of images of head, torso, and leg parts. With these image pairs, we extracted the image features and recognized individuals by measuring the distance between images. As shown in our previous experimental results in Section 3.2.2 and Section 3.2.3, the CNN-based feature extraction method was proven to outperform the HOG and MLBP feature extraction methods. Therefore, in this experiment, we used the CNN-based feature extraction method to extract the image’s features. The detailed experimental results are shown in Table 8. In addition, we measured the CMC curves of the part-based identification systems, and the results are shown in Figure 17, Figure 18 and Figure 19. Compared to results in Figure 11 and Figure 14, we can see that the identification results of systems that use body parts are worse than those of the systems that use the full-body images. These results are caused by the fact that the identity information in each body-part is less than the full body. As shown in Table 8 and these figures, the systems that use the torso part of the human body produced the best verification results among the three parts, whereas the systems that use the leg part produced the worst verification results. In detail, using the head part, we obtained an EER of 9.875%; using the torso part, we obtained an EER of 5.995%; and using the leg part, we obtained the lowest verification result of 18.375%. Through these results, we can conclude that the torso part contains more identity information than the head or leg part.

**Figure 20:**
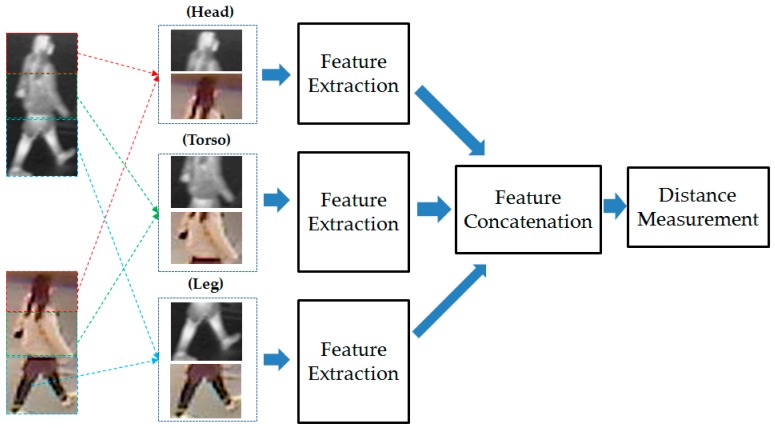
Flowchart of gender recognition using the combination of different body parts.

As our final experiment, we performed the gender recognition by combining the extracted features from different parts of human body (i.e., head, torso and leg part) to exploit the recognition ability of this scheme compared to the use of the entire human body images. In Figure 20, we show the flow chart of this experiment. Then, the detailed experimental results of this experiment are shown in Table 9. Compared to the results in Table 8 where the recognitions were performed using separated human body parts, i.e., head, torso or leg parts, this experiment produced the better recognition results in both cases of using Euclidean distance and correlation distance. In detail, the best recognition error (EER) in this experiment is 5.265% that is smaller than the errors in Table 8 (with EER of 9.875% using head part, 5.995% using torso part and 18.375% using leg part). However, this result is worse than the recognition result that uses the whole body image for recognition in Table 7 (with EER of 1.465%).

This result is caused by the fact that the human body has very big variation as explained in Section 1. The variation could appear larger during the movement of human body. As the result, some parts of human body such as leg or head parts have larger variation than the other parts. The appearance of large variation of a specific body part makes the recognition fail on this part. In contrast, using the entire human body image the CNN can try to learn the invariant features while reduces the effect of large variation parts (variant and invariant parts are included in the training images). From the result of this experiment, we see that the recognition should be done using the entire human body image instead of using the combination of separated parts.

### 3.3. Discussion

In a biometrics, conventional recognition system can operate in two modes: verification mode and identification mode. The difference between the two modes is that the verification mode performs the one-by-one matching, whereas the identification mode performs the one-by-n matching. Because our system for person recognition can be primarily used in surveillance environment, we mainly consider the case that our system is used for identification, and measured the identification accuracies as shown in Figure 12, Figure 15 and Figure 17, Figure 18 and Figure 19. However, as shown in these figures, identification rate according to rank cannot shows the FAR (the error rate of incorrectly accepting unenrolled person as enrolled one), and only the correct recognition rate (CRR) (the rate of correctly accepting enrolled person as enrolled one) can be measured. These two error rates of FAR and FRR (100–CRR (%)) have the trade-off characteristics. Larger FAR causes smaller FRR where smaller FAR does larger FRR. Therefore, we additionally measured the verification accuracies in terms of EER and ROCs in order to consider both FAR and FRR in Section 3.2.2, Section 3.2.3 and Section 3.2.4.

The extracted image features in our study are histogram-like features (HOG, MLBP or CNN features). For similarity measurement, as mentioned in our paper, the Euclidian and correlation distance have been widely used by previous researches [25,57,58,59]. Beside these two measurement methods, other methods can be used. For example, Lee et al. [7] used the chi-square distance to measure the similarity degree of face images for face-based person recognition. However, this method cannot be used in our research because it requires the non-negative input features. As shown in our paper, the extracted image features were firstly transformed to PCA domain to reduce the feature dimension and effect of noise. As a result, the final features that are used for similarity measurement can contain negative components that cannot be used for chi-square method. Therefore, we use two common distance measurement methods of Euclidean and correlation in our research.

As shown in our paper, the combination of human body images in two bands (visible and IR band) is efficient for enhancing the recognition performance. However, the cost of the equipment (visible and thermal cameras) and the processing time are higher than the case of using only visible or thermal band. In contrast to the use of only visible light images, our proposed method requires to use an additional thermal camera. In addition, it could also require additional graphic processing unit (GPU) for CNN processing. Therefore, the cost of equipment is increased compared to previous systems that use only visible light or thermal image for recognition. Nevertheless, recently, the deep learning method is fast developing for image-based systems. One of the motivations for this development is the appearance of GPU which are dedicated for processing a huge amount of arithmetic operations in parallel. Using GPU, the processing time is much reduced. In addition, as the development of technology and the requirement of high level of reliability of systems, the cost of thermal camera and GPU has been rapidly reduced, which can make them feasible to be adopted in various applications.

As a result of our study, we conclude that the combination of visible light and thermal images of human body can be used to enhance the performance of the body-based person recognition system. In addition, the use of CNN method for feature extraction of visible light and thermal images of human body is more sufficient than hand-designed methods such as HOG or MLBP. These results can be applied in the real-world surveillance systems which use the combination of visible light and thermal camera to enhance the management ability of traditional surveillance systems. In our experiments, we captured the images of human body in outdoor (uncontrolled) environment to simulate the operation of real-world systems. Therefore, we can find that the experimental results reflect the real operation of surveillance systems.

## 4. Conclusions

In this paper, we proposed a method for person recognition in a surveillance system environment using a combination of visible light and thermal images of the human body. In our research, identity information from the human body was captured using two different kinds of cameras, a visible light and a thermal camera. Inspired by recent research in computer vision, a CNN was employed to extract the optimal features from the input images. Through experimental results by various system configurations, we confirmed that the recognition accuracy of the proposed method that uses a combination of visible light and thermal images of the human body was superior to those of the systems that use only single visible light or single thermal images for the recognition problem. In addition, the CNN is more suitable for image features extraction for the recognition system than the HOG and MLBP methods.

## Figures and Tables

**Table 1:** Summary of previous and proposed studies on person recognition (verification/identification) using body images.

Category	Method	Strength	Weakness
Using only visible light images of the human body for the person identification problem	Extracts the image features by using traditional feature extraction methods such as color histogram [17,18], local binary pattern [17,18], Gabor filters [19], and HOG [19].	Easy to implement image features extraction by using traditional feature extraction methods [17,18,19].	- The identification performance is strongly affected by random noise factors such as background, clothes, and accessories. - It is difficult for the surveillance system to operate in low illumination environments such as rain or nighttime because of the use of only visible light images.
	- Uses a sequence of images to obtain body gait information [23,24].	- Higher identification accuracy than the use of single images [23,24].	
	- Uses deep learning framework to extract the optimal image features and/or learn the distance measurement metrics [14,15,25].	- Higher identification accuracy can be obtained; the extracted image features are slightly invariant to noise, illumination conditions, and misalignment because of the use of deep learning method [14,15,25].	
Using a combination of visible light and thermal images of the human body for the person verification and identification problem (our proposed method)	- Combines the information from two types of human body images (visible light and thermal images) for the person verification and identification problem. - Uses CNN and PCA methods for optimal image features extraction of visible light and thermal images of human body.	- Verification/identification performance is higher than that of only visible light images or only thermal images. - The system can work in poor illumination conditions such as rain or nighttime.	- Requires two different kinds of cameras to acquire the human body images, including a visible light camera and a thermal camera. - Requires longer processing time than the use of a single kind of human body image.

**Table 2:** Detailed structure description of our proposed CNN method for the person recognition problem. (M is the number of individuals in the training database; n/a—not available).

Layer Name	No. Filters	Filter Size	Stride	Padding	Output Size
Input Layer	n/a	n/a	n/a	n/a	115 × 179 × 1
Convolutional Layer 1 & ReLU (C1)	96	7 × 7	2 × 2	0	55 × 87 × 96
Cross-Channel Normalization Layer	n/a	n/a	n/a	n/a	55 × 87 × 96
MAX Pooling Layer 1 (C1)	n/a	3 × 3	2 × 2	0	27 × 43 × 96
Convolutional Layer 2 & ReLU (C2)	128	5 × 5	1 × 1	2 × 2	27 × 43 × 128
Cross-Channel Normalization Layer	n/a	n/a	n/a	n/a	27 × 43 × 128
MAX Pooling Layer 2 (C2)	n/a	3 × 3	2 × 2	0	13 × 21 × 128
Convolutional Layer 3 & ReLU (C3)	256	3 × 3	1 × 1	1 × 1	13 × 21 × 256
Convolutional Layer 4 & ReLU (C4)	256	3 × 3	1 × 1	1 × 1	13 × 21 × 256
Convolutional Layer 5 & ReLU (C5)	128	3 × 3	1 × 1	1 × 1	13 × 21 × 128
MAX Pooling Layer 5 (C5)	n/a	3 × 3	2 × 2	0	6 × 10 × 128
Fully Connected Layer 1 & ReLU (FC1)	n/a	n/a	n/a	n/a	4096
Fully Connected Layer 2 & ReLU (FC2)	n/a	n/a	n/a	n/a	2048
Dropout Layer	n/a	n/a	n/a	n/a	2048
Fully Connected Layer 3 (FC3)	n/a	n/a	n/a	n/a	M

**Table 3:** Description of the training and testing databases in our experiments.

Database		Males	Females	Total
Training Database	Number of Persons	204 (persons)	127 (persons)	331 (persons)
	Number of Original Images	4080 images (204 × 20)	2540 images (127 × 20)	6620 (images)
	Number of Artificial Images	20400 images (204 × 20 × 5)	10160 images (127 × 20 × 5)	33100 images
Testing Database	Number of Persons	50 (persons)	31 (persons)	81 (persons)
	Number of Original Images	1000 images (50 × 20)	620 images (31 × 20)	1620 images
	Number of Artificial Images	5000 images (50 × 20 × 5)	3100 images (31 × 20 × 5)	8100 images

**Table 4:** Verification performance (EER) of the recognition system using Euclidean distance without applying PCA on extracted image features (unit: %).

Feature Extraction Method	Using Only Visible Light Images	Using Only Thermal Images	Using Combination of Visible Light and Thermal Images
HOG [19]	12.085	13.905	11.055
MLBP [17,18]	13.735	16.695	12.775
CNN	7.315	6.815	4.285

**Table 5:** Verification accuracy (EER) of the recognition system using Euclidean distance with PCA applied on extracted image features (unit: %).

Feature Extraction Method	Using Only Visible Light Images	Using Only Thermal Images	Using Combination of Visible Light and Thermal Images
HOG [19]	10.665	12.015	8.915
MLBP [17,18]	13.485	17.025	12.535
CNN	6.295	5.745	2.945

**Table 6:** The verification accuracy (EER) of the recognition system using correlation distance without applying PCA on extracted image features (unit: %).

Feature Extraction Method	Using Only Visible Light Images	Using Only Thermal Images	Using Combination of Visible Light and Thermal Images
HOG [19]	11.595	12.655	10.125
MLBP [17,18]	11.105	12.855	9.885
CNN	4.775	3.185	**1.645**

**Table 7:** Verification accuracy (EER) of the recognition system using correlation distance with PCA applied on the extracted image features (unit: %).

Feature Extraction Method	Using Only Visible Light Images	Using Only Thermal Images	Using Combination of Visible Light and Thermal Images
HOG [19]	7.355	6.635	5.265
MLBP [17,18]	6.995	8.125	5.395
CNN	4.215	2.905	**1.465**

**Table 8:** Verification performance (EERs) of the systems that use body parts for the recognition (unit: %).

Body Part	Distance Method	PCA Method	Using Only Visible Light Images	Using Only Thermal Images	Using Combination of Visible Light and Thermal Images
Head	Euclidean Distance	Without PCA	20.494	17.145	16.064
		With PCA	19.265	16.585	14.725
	Correlation Distance	Without PCA	18.485	17.605	14.875
		With PCA	14.985	13.335	**9.875**
Torso	Euclidean Distance	Without PCA	17.654	12.465	10.815
		With PCA	16.465	11.845	9.755
	Correlation Distance	Without PCA	14.695	10.684	7.925
		With PCA	11.515	8.905	**5.995**
Leg	Euclidean Distance	Without PCA	22.454	25.134	20.025
		With PCA	23.145	25.895	21.235
	Correlation Distance	Without PCA	24.224	26.505	22.305
		With PCA	20.705	23.675	**18.375**

**Table 9:** Verification performance (EERs) of the system that combines features from different parts of human body for recognition (unit: %).

Distance Methods	Using Only Visible Images	Using Only Thermal Images	Using Combination of Visible and Thermal Images
Using Euclidean Distance	13.724	11.915	9.165
Using Correlation Distance	9.155	8.405	**5.265**
